# Degradation kinetics of artesunate for the development of an ex-tempore intravenous injection

**DOI:** 10.1186/s12936-022-04278-4

**Published:** 2022-09-06

**Authors:** Fanta Gashe, Evelien Wynendaele, Bart De Spiegeleer, Sultan Suleman

**Affiliations:** 1 Jimma University Laboratory of Drug Quality (JuLaDQ) and School of Pharmacy, Jimma University, PO Box 378, Jimma, Ethiopia; 2 Drug Quality and Registration (DruQuaR) Group, Faculty of Pharmaceutical Sciences, Ghent University, Harelbekestraat 72, 9000 Ghent, Belgium

**Keywords:** Intravenous formulations, Ex-tempore, Artesunate, Stability, Kinetics, Optimization

## Abstract

**Background:**

Artesunate is recommended by the World Health Organization (WHO) for parenteral treatment of severe *Plasmodium falciparum* malaria. However, artesunate is inherently unstable in an aqueous solution and hydrolyses rapidly after its preparation for injection. Therefore, the aim of the study was to evaluate the stabilizing effects of phosphate buffer and mannitol against short-term (ex-tempore) artesunate hydrolysis.

**Methods:**

A HPLC–UV isocratic method was developed using a reversed-phase fused core column (HALO RP-C18) and a mobile phase consisting of a mixture of 45% ammonium formate 10 mM in water (pH 4.5) and 55% methanol. Artesunate was formulated as aqueous solutions using a design of experiment (DOE) to investigate the artesunate stabilizing effects of pH (8–10), phosphate buffer strength (0.3–0.5 M), and mannitol (0–0.22 mmol/mL). The solutions were incubated at predefined temperatures (5, 25, and 40 °C) with subsequent analysis. Arrhenius equation was applied to model and evaluate the stability results.

**Results:**

The developed HPLC-based method using fused-core stationary phase allowed to selectively quantify artesunate in the presence of its main hydrolysis degradants; namely β-dihydroartemisinin (β-DHA) and α-dihydroartemisinin (α-DHA) within 10 min. By applying the Arrhenius equation, the rate of hydrolysis of the drug increased approximately by 3.4 as the temperature raised by 10 °C. Buffer strength was found to be the main factor affecting the hydrolysis rate constants at 5 and 25 °C (p < 0.05), the activation energy (p = 0.009), and the frequency factor (p = 0.045). However, the effect of the buffer was predominant on the activation energy and hydrolysis rate constants, revealing its stabilizing effect on the drug at lower buffer strength (0.3 M). Within the investigated range (pH = 8–10), pH was found to influence the activation energy, with a positive stabilizing effect in the pH range of 8–9. The addition of mannitol as stabilizing agent into artesunate aqueous formulation did not show an improved response.

**Conclusion:**

Phosphate buffer was the main stability determining factor of artesunate in the aqueous intravenous (i.v.) formulation and was found to be more effective in stabilizing artesunate at a buffer strength of 0.3 M in pH 8–9, while mannitol lacked stabilizing effect.

**Supplementary Information:**

The online version contains supplementary material available at 10.1186/s12936-022-04278-4.

## Background

Artesunate is a semisynthetic compound currently applied for the treatment of malaria using various pharmaceutical forms [1, 2]. Moreover, it has a broad therapeutic potential, such as anticancer activity [3–7] as well as inhibitory effects against schistosomiasis [8], leishmaniasis [9], *Mycobacterium tuberculosis* [10], and viral infections [11, 12].

**Fig. 1:**
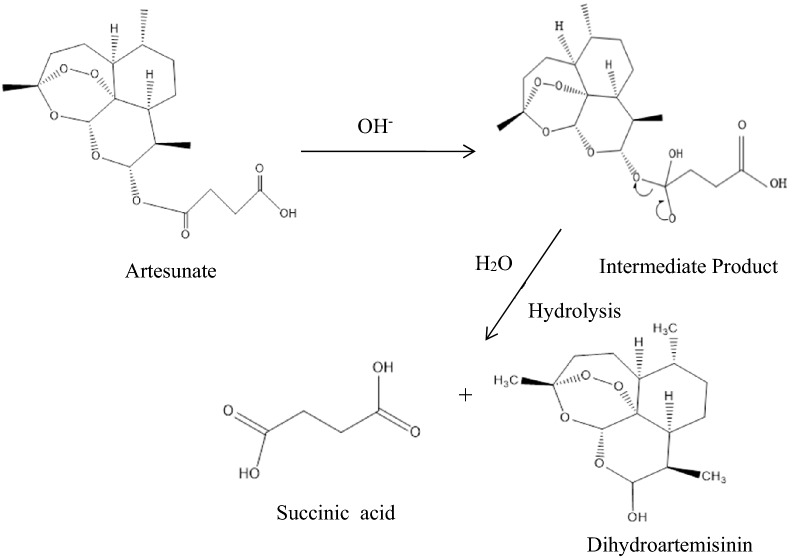
Base-catalysed hydrolysis pathway of artesunate in water

According to the 2021 guideline for malaria, World Health Organization (WHO) recommends parenteral treatment with artesunate for severe *Plasmodium falciparum* malaria, which is the deadliest malaria parasite, especially in Africa, in preference to quinine [13]. Intravenous artesunate has been shown to significantly reduce the risk of death, associated with a lower risk of hypoglycaemia. It is relatively safe in comparison to quinine with no serious drug-related adverse effects [14, 15]. However, artesunate is a hemisuccinate ester wherein the hemiacetal OH group of dihydroartemisinin is acylated with succinic acid. It is unstable and rapidly hydrolysed to its active metabolite dihydroartemisinin [16–18] (see Fig. 1).

The degradation of artesunate can be dependent on the formulation compositions [19–21]. Hence, stable aqueous formulations for parenteral use, which are physiologically compatible, are currently not available. Nowadays, a common practice for the preparation of intravenous injections of artesunate is by dissolving the sterile drug substance powder in an alkaline aqueous solvent (sodium carbonate). Further dilution is then done using physiological saline of buffer just prior to administration of the drug [22]. The WHO recommends that this product should be freshly prepared and administered within 1.5 h [13, 23]. Therefore, there is still a need for further development of artesunate aqueous parenteral formulations with improved chemical stability.

The stability of parenteral products can be improved by their formulation composition, i.e. by their pharmaceutical excipients [24, 25]. Mannitol has been a widely used additive as a stabilizing and bulking agent in parenteral formulations owing to its excellent stability in aqueous solution, and extensive compatibility [26, 27]. Phosphate buffer has a wide range of buffering capacity and safety, hence commonly added to injectable products to control the pH, resist unwanted pH changes, and retard the degradation of the drugs by its pH and buffer effects [27, 28]. Therefore, in this study, a new artesunate formulation, which was as simple and economic as possible, was developed using a disodium phosphate buffer and mannitol, and their effect as stabilizing agents was examined with the aim of delaying the hydrolysis of the vulnerable ester bond.

The rate of drug hydrolysis depends not only on the composition of the formulation but also on storage temperature [29]. The quantitative relationship between the rate of a chemical reaction and the temperature can be modelled by the Arrhenius equation. According to Arrhenius kinetics, the higher the activation energy and the lower the frequency factor, the less hydrolysis of the ester-linkage occurs [30]. As a result of this relationship, the extrapolated data from the Arrhenius plot were used to predict the effects of the buffer strength, pH, and mannitol concentration on the stability of artesunate in aqueous solution. Thus, a stability-indicating HPLC method for the quantification of artesunate was first developed with subsequent validation. Then, the stability of artesunate following the in-situ preparation of different injectable formulations using a Design of Experiment (DOE) approach was investigated, leading to a proposed best parenteral formulation.

## Methods

### Chemicals

Artesunate drug substance was obtained from IPCA (Mumbai, India) with purity above 96.0% complying with the International Pharmacopoeia. HPLC gradient grade methanol was purchased from Fisher Scientific (UK). An Arium 611 purification system of Sartorius (Germany) was used to purify water at 18.2 MΩ cm quality. Disodium phosphate was obtained from Merck (Germany). Formic acid (Riedel-de Haën, Germany), mannitol (Sigma-Aldrich, USA), ammonium formate and phosphoric acid (Fluka, Switzerland) were all analytical grades.

### Method development and validation

#### HPLC method

HPLC–UV analyses were carried out on a Waters Alliance 2695 separation module equipped with a Waters 2487 Dual λ absorbance detectors (all Waters, USA). The artesunate content of the products was determined at 210 nm. Data handling was performed with Empower 2 software. In a standard HPLC run, 10 μL was injected and separated on a HALO RP-C18 column (50 × 4.6 mm, 1.7 µm solid core particle with a 0.5 mm porous silica layer fused to the surface) at a flow rate of 1.1 mL/min (Advanced materials technology, Wilmington, USA). An isocratic LC separation with a mobile phase consisting of a mixture of 45% (A) 10 mM m/V ammonium formate in water at pH 4.5, and 55% (B) methanol, was performed maintaining the column at 25 ± 5 °C.

#### Preparation of reference solutions

A stock solution was prepared by dissolving the accurately weighed 400 mg of artesunate in 50 mL 45/55% *V/V* 10 mM ammonium formate/MeOH. The solution was further diluted with mobile phase to prepare in a range of 10–120% label claim (l.c.). A 100% reference solution corresponds to 3.33 mg/mL artesunate.

#### Sample preparation

Samples of artesunate aqueous solution (250 µL) from each formulation containing mannitol and phosphate buffer were taken. The solutions were then centrifuged at 17,000*g* for 4 min at 5 °C to precipitate the phosphate in order to protect the silica-based analytical column and thus prolong its lifetime. Subsequently, the supernatant was collected and diluted with methanol and then acidified with 0.1 M formic acid to obtain a 100% label claim corresponding to 3.33 mg/mL in 50/50% V/V MeOH/purified water acidified with formic acid.

#### Validation procedure

Prior to the validation process, the analytical HPLC method suitability test was carried out by injecting six replicates of the standard sample (3.33 mg/mL). The number of theoretical plates, resolution, and tailing factors were assessed. Subsequently, the newly developed HPLC method was validated for specificity, linearity and range, accuracy, precision, the limit of detection (LoD), and limit of quantitation (LoQ) in accordance with ICH guidelines (Q2A and Q2B).

#### Specificity

The presence of hydrolytic products, β-DHA, and α-DHA, close to artesunate is critical during its analysis. Thus, the specificity was determined in the presence of the matrix and related compounds, i.e. a placebo, a placebo spiked sample, and a spiked placebo with DHA.

#### Linearity and range

Serial artesunate solutions were prepared and evaluated over the concentration range of 10–120% l.c. The calibration curve was constructed, and then the correlation coefficient, y-intercept, and the slope of the regression line were obtained.

#### Accuracy

Accuracy was determined by spiking artesunate reference solution into the placebo samples. It was established across the specified range of the analytical procedure at 10, 50, and 100% l.c. with 3 replicated injections of each spiked placebo.

#### Repeatability/precision

Repeatability and intermediate precision of the method were investigated by injecting the sample solutions in triplicate at three concentration levels (3.33 mg/mL, 1.66 mg/mL, and 0.33 mg/mL) consisting of a total of 9 and 18 chromatogram runs, respectively. The RSD of the assay results was calculated at each concentration level.

#### Limit of detection (LoD) and limit of quantitation (LoQ)

LoD and LoQ were calculated out of the calibration curve by using the following equations:

1 $${\text{LoD}} = \frac{{3.3*\sigma }}{{\text{S}}}\,\,{\text{and}}{\mkern 1mu} \,{\text{LoQ}} = \frac{{10*\sigma }}{{\text{S}}},$$

σ is the standard deviation of the y-intercept of the regression line while S is the slope of the calibration curve of the analyte.

#### Artesunate product development for degradation kinetics study

Artesunate (40 mg/mL) was dissolved in the aqueous solution of phosphate buffer with or without mannitol according to the experimental design (Additional file 1: Table S1). Each formulated artesunate solution was incubated at predefined temperatures in a Max Q 4000 (Thermo Scientific, San Jose, USA) at 25 and 40 °C and in a refrigerator at 5 °C. Aliquots of artesunate solutions were withdrawn at different intervals, and assayed as described earlier.

#### Kinetic evaluation of the hydrolysis of artesunate

The kinetics were determined by taking as an example the artesunate solution containing 0.3 M phosphate buffer at pH of 9 in presence of 0.22 mmol/mL mannitol. At the three different temperatures experimentally explored, the hydrolysis kinetics, and the observed rate constants k were determined. Arrhenius equation (using Eq. (2)) was applied to determine the activation energy and the frequency factor, as well as to calculate the half-life (t_1/2_), and the shelf-life (t_0.9_) of the formulation.

2 $$\mathrm{ln k}=\mathrm{ln A}-\frac{\mathrm{Ea}}{\mathrm{RT}},$$

where k = specific rate constant, Ea = activation energy, A = frequency factor T = temperature (Kelvin).

In addition, the effect of buffer strength on the degradation rate of artesunate in aqueous solution was evaluated at different pH values and temperatures. A Design of Experiments (DOE) approach was hence applied using three controlled independent variables, i.e. buffer strength, pH, and mannitol concentration. The former two variables were set at 3 levels while the last variable was studied at 2 levels. The investigated levels are given in Table 1. Hydrolysis rate constants, the activation energy, and frequency factor were selected as dependent factors or responses to finally model the stability of the drug. A total of 18 experimental units were thus executed in a random sequence to minimize uncontrolled influences on the estimated effects (Additional file 1: Table S1).

**Table 1:** Factors and their levels investigated

Factor	Levels		
	− 1	0	+ 1
(A) Disodium phosphate buffer strength (mmol/mL)	0.30	0.40	0.50
(B) pH	8	9	10
(C) Mannitol concentration (mmol/mL)	0		0.22

#### Statistical analysis and optimization

Statistical analysis and the least-squares linear regression were performed using Stata/SE Statistics for Windows, version 14, including manual Dixon and Grubbs’ tests for detection of outliers. After building the model, it was interpreted graphically by visualizing 2D contour plots and 3D response surface plots for each response using the Design-expert version 13 (USA). Furthermore, the model was interpreted statistically by the determination of the significance of the factors.

## Results

### Method development and validation

Various analytical parameters were examined to optimize the separation of artesunate from its degradants as well as to have an acceptable sufficient peak symmetry for quantification. Two columns were evaluated, i.e. RP C18 Halo^®^, 4.6 × 50 mm, and RP amide Halo^®^, 4.6 × 50 mm at various mobile phase compositions. The RP amide column did not give sufficient resolution between artesunate and DHA under all conditions investigated. The optimized HPLC method was developed by using the RP C18 HALO^®^ column. It was found to be suitable for the determination of artesunate in the presence of its degradation products (α-DHA and β-DHA) using the mobile phase 10 mM ammonium formate (45%) at pH 4.5 and methanol (55%) with a high and robust resolution (i.e. 8.4) as well as peak symmetry (i.e. 1.6), which complies with pharmacopoeial specifications.

### Validation

#### Specificity

**Fig. 2:**
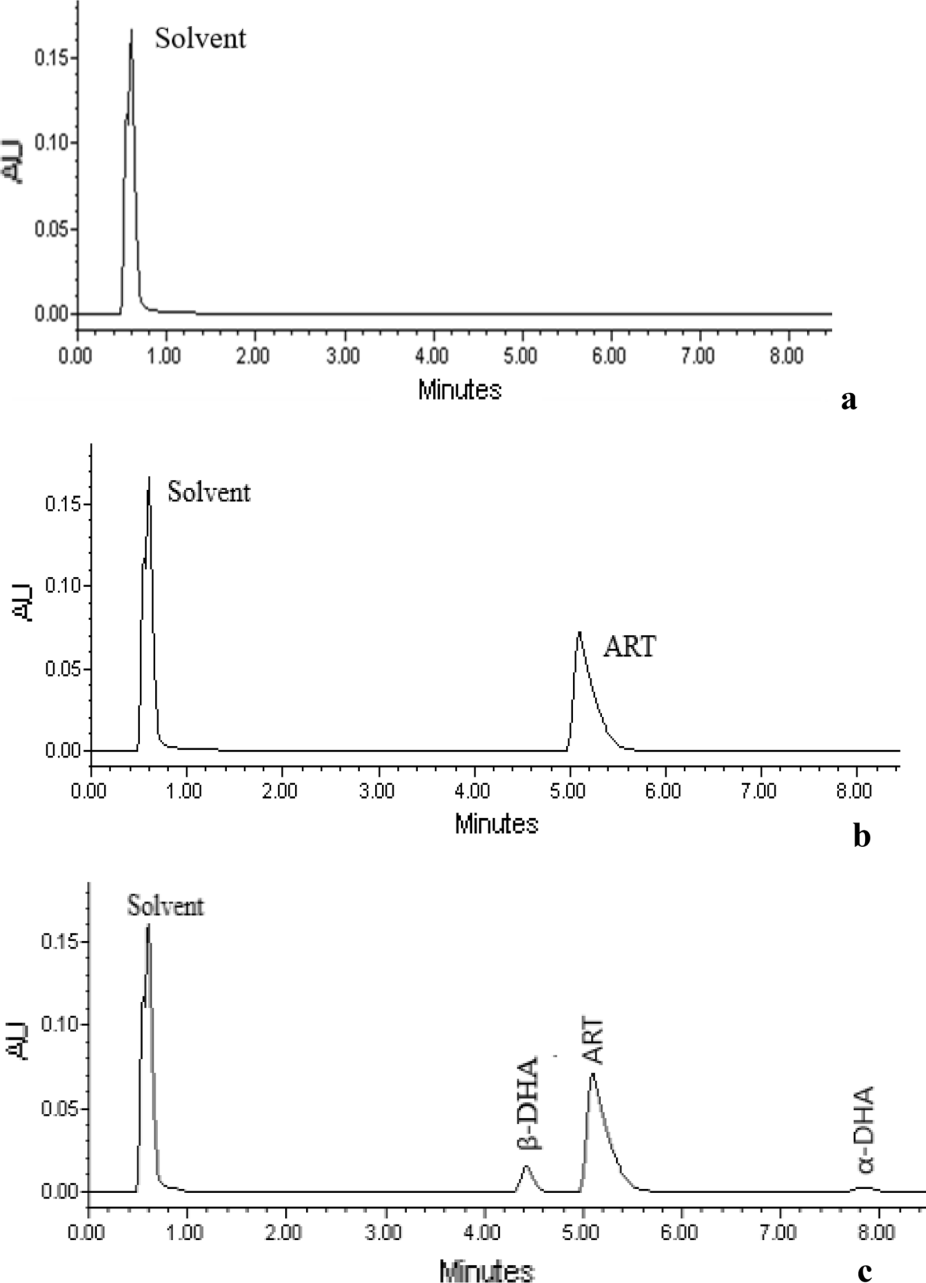
Artesunate (ART) in mannitol containing phosphate buffer (**a** placebo, **b** spiked placebo, **c** artesunate with degradants β-DHA and α-DHA)

The presence of DHA in artesunate solution, which is its main hydrolytic degradative product, did not interfere with the analysis of artesunate. The chromatograms of dihydroartemisinin isomers were well separated from artesunate, showing that the method is specific. The results are illustrated in Fig. 2.

#### Linearity

Twelve different concentrations of artesunate solution containing the formulation components such as mannitol and phosphate buffer were employed to set up linearity. The relationship between the peak area and the concentration was linear (0.9974) in the assayed range of 10–120% label claim, and the result is illustrated in Table 2.

**Table 2:** Calibration curve evaluation of the chromatographic method

Regression parameters	Values
Regression coefficient, R^2^	0.9974
Slope ± standard error	439.72 ± 7.11
Intercept ± standard error	− 35.68 ± 17.45
Relative standard error (%)	1.6
F-value	3819.83
Concentration range (mg/mL)	0.33–4
Residual standard error	28.37
Number of points	12

#### Accuracy

Recovery experiments showed no significant loss of artesunate due to the sample treatment. The mean recovery of the drug from spiked samples was 103.8% (RSD = 1.8). In the concept of quality-by-design, the relatively small inaccuracy of the newly-developed HPLC method is not a major hurdle and will not restrain the method from its application in early-phase formulation development.

#### Repeatability/precision

The variability of the results obtained for three spiked placebos on the same day (nine analyses) was low with a % RSD of 2.4, 1.37, and 0.62 for the label claim of 10%, 50%, and 100%, respectively. Similarly, the relative standard deviations for intermediate precision were 2.11, 2.81, and 2.93 at concentrations of 0.33, 1.67, and 3.33 mg/mL solutions, respectively, indicating that the method is within the acceptance criteria of 5%. Hence, the method is sufficiently precise.

#### LoD and LoQ

By using Eq. (2), LoD and LoQ were calculated to be 0.131 mg/mL and 0.397 mg/mL, respectively. Both limits were determined based on the standard deviation and the slope of the calibration curve.

### Kinetic evaluation of the stability of artesunate

**Fig. 3:**
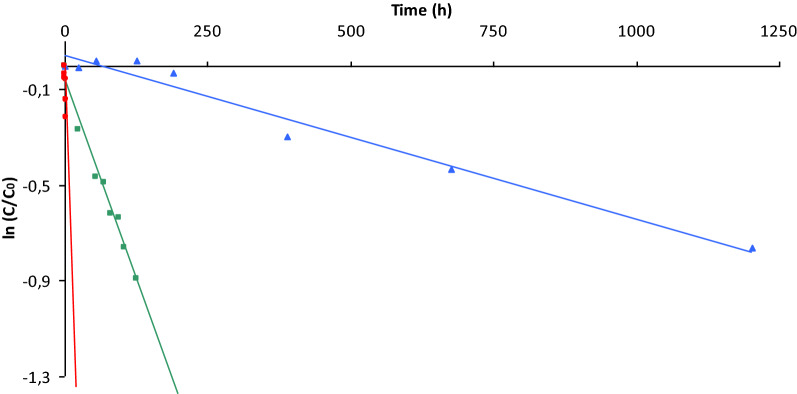
First-order plots showing the degradation of artesunate (0.3 M phosphate, pH 9, 0.22 mmol/mL mannitol) at various temperatures: (filled circle) 313.15 k, (filled rectangle) 298.15 k, (filled triangle) 278.15 k

The results of the degradation kinetics of artesunate in aqueous solution formulated with disodium phosphate and mannitol revealed the correlation coefficients of the semi-logarithmic plots of ln (percentage concentration drug remaining) versus time > 0.98. This suggests the ester hydrolysis of artesunate followed pseudo-first order kinetics over the temperature range of 5 °C to 40 °C (Fig. 3). The estimated degradation rate constants (k) were 0.056 h^−1^, 0.006 h^−1^, and 0.00066 h^−1^ with the estimated half-life (t_0.5_) of 12.4 h, 114.6 h, and 1053.4 h at 40, 25, and 5 °C. The rate of hydrolysis of the drug was predicted to increase approximately by 3.4 folds as the storage temperature of the formulation raised by 10 ℃ (Table 3).

**Table 3:** Degradation kinetics of artesunate

Temperature (K)	k (h^−1^)	Half-life (t_o.5_) h	Shelf life (t_0.9_) h
313.15	5.6 × 10^–2^ ± 6 × 10^–3^	12.4 ± 1.2	1.9 ± 0.2
298.15	6.1 × 10^–3^ ± 3 × 10^–4^	114.6 ± 5.4	17.4 ± 0.8
278.15	6.6 × 10^–4^ ± 4 × 10^–5^	1053.4 ± 59.9	160.1 ± 9.2

**Fig. 4:**
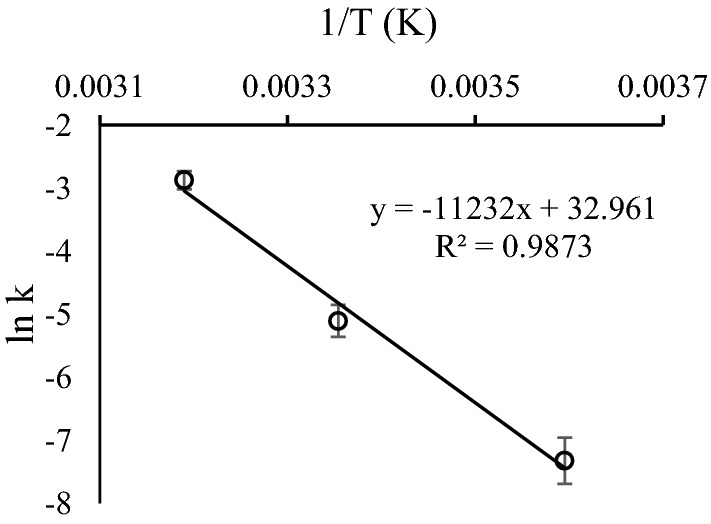
The Arrhenius plots of artesunate (0.3 M phosphate, pH 9, 0.22 mmol/mL mannitol)

The hydrolysis of artesunate fits the Arrhenius relationship within the investigated temperature range (r^2^ > 0.98) (Fig. 4). The estimated values of activation energy and frequency factor from the Arrhenius equations for artesunate in presence of mannitol (0.22 mmol/mL) and phosphate buffer (0.3 M) were 90.84 kJ/mol and 6.67E + 13 h^−1^, respectively. Interpolating using this equation, artesunate had a half-life (t_0.5_) of 47.4 h or 1.97 days and a shelf-life (t_0.9_) of 7.2 h at 30 °C, a temperature representative of ambient conditions in tropical countries. Similarly, the Arrhenius plot was applied to all experiments to estimate the activation energy, which is presented in Additional file 1: Table S2.

### Modelled effects of the three variables

Liner and 2FI models were used for the evaluation of the influences of the factors over the investigated levels on the hydrolysis rate constants of the drug. For the k at 5 ℃, the 2FI model with r^2^ (0.7297) and a significant F-value of 4.5 (p = 0.0182) was applied. The adequate precision which is the measure of signal to noise ratio of the model was 7.724, greater than the minimal desirable value, 4. Similarly, a linear model which was found to fit the data at 25 °C with r^2^ 0.6259 and F- value of 7.25 (p = 0.0042) was selected. The equations for the degradation rate constants are given below.

3 $${\text{k}}\left( {{\text{at}}\,{\text{5}}\,^\circ{\text{C}}} \right) = -\,0.005 + 0.0113{\text{X}}_{1} + 0.0007{\text{X}}_{2} + 0.0068{\text{X}}_{3} - 0.0013{\text{X}}_{1} {\text{X}}_{2} + 0.0095{\text{X}}_{1} {\text{X}}_{3} - 0.0012{\text{X}}_{2} {\text{X}}_{3} ,$$

4 $${\text{k}}\left( {{\text{at 25 }}\,^\circ {\text{C}}} \right) = 0.0039 + 0.0043{\text{X}}_{1} + 6.9 \times 10{\text{exp}}\left( { - 06} \right){\text{X}}_{2} + 0.0026{\text{X}}_{3} ,$$

X_1_, X_2_, X_3_ are dependent variables (X_1_ = buffer strength, X_2_ = pH, X_3_ = mannitol concentration); k is hydrolysis rate constant.

**Fig. 5:**
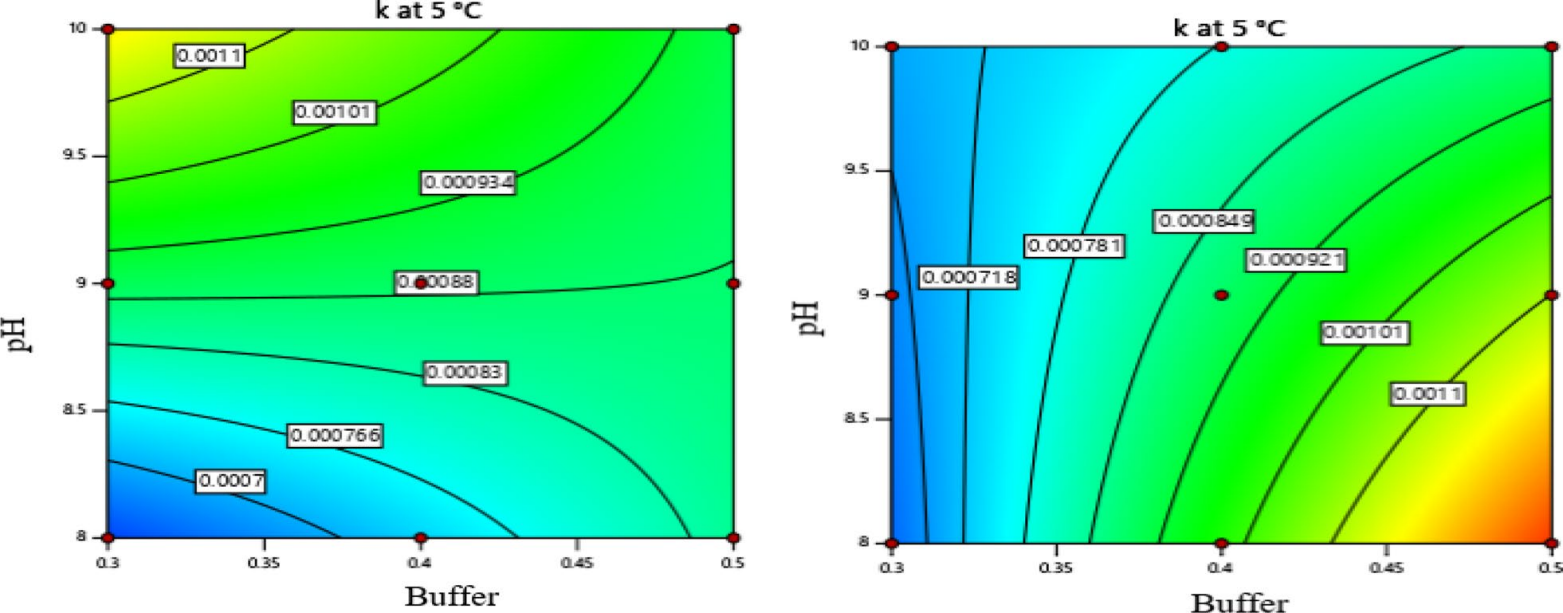
Counter plot of degradation rate constant of artesunate at 5 ℃ at low (left) and high (right) level of mannitol

**Fig. 6:**
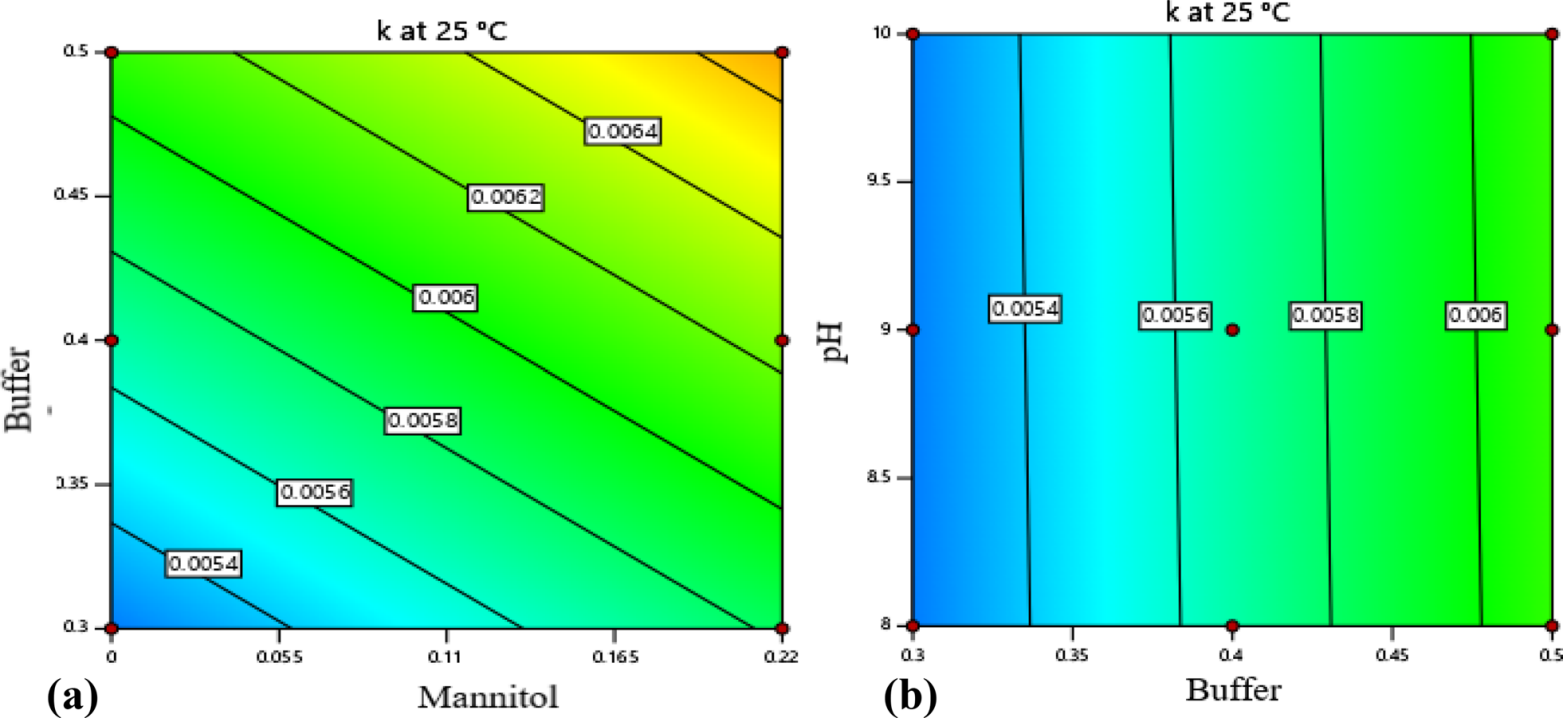
Counter plot of degradation rate constant of artesunate at 25 ℃

The values for the effect of the independent factors in all artesunate formulations on the degradation rate constants and Arrhenius parameters were presented in Additional file 1: Table S2. The hydrolysis rate of the drug was found to be affected significantly by buffer strength at 5 °C and 25 °C (p < 0.05) (Additional file 1: Tables S3, S4). At both temperature levels, the degradation rate of the drug was accelerated at a higher concentration of the buffer (0.5 M). The interaction effect of the factors was also observed at both low and high levels of mannitol as illustrated in Fig. 5. The rate constants increased as the buffer concentration raised from 0.3 M to 0.5 M, and the pH of the formulation shifted from 8 to 10 at 5 °C. However, a significant variation of the pH influences was not observed at 25 ℃ as shown in Fig. 6b. The presence of mannitol in the formulation did not decrease the degradation rate of the drug at all temperatures. The hydrolysis rate was even significantly increased at a high level of mannitol with a concentration level of 0.22 mmol/mL at 25 °C (p < 0.05) (Fig. 6a). On the other hand, the hydrolysis rate of the drug was not significantly varied at 40 ℃ over the experimented levels of the factors (p > 0.05) (Additional file 1: Table S5).

The quadratic model (Eq. 5) was selected for activation energy with R-Square, Adjusted R-Square, and PRESS values of 0.9185, 0.8371, and 268.42, respectively. The model was found to be significant (p = 0.0013) with F-value of 11.28, showing that there was only 0.13% probability of F- value of this large which could occur due to noise. The influences of the factors on frequency factor were also evaluated using the linear model, which was found to fit with F-value of 5.34 (p = 0.0355). Following models resulted:

5 $${\text{Ea}} = -122.23-402.68{\text{X}}_{1} + 67.92{\text{X}}_{2} - 203.76{\text{X}}_{3} + 38.84{\text{X}}_{1} {\text{X}}_{2} - 110.76{\text{X}}_{1} {\text{X}}_{3} + 26.53{\text{X}}_{2} {\text{X}}_{3} + 47.97{\text{X}}_{1}^{2} - 4.80{\text{X}}_{2}^{2},$$

6 $$\mathrm{A}= 6.6 \times 10\mathrm{exp}\left(13\right)-1.34 \times 10\mathrm{exp}\left(14\right)\mathrm{X}_1,$$

X_1_, X_2_, X_3_ are dependent variables (X_1_ = buffer strength, X_2_ = pH, X_3_ = mannitol concentration); Ea is activation energy, and A is frequency factor.

Variance analysis revealed that the effects of all the main terms, the interactions except buffer with mannitol, and the quadratic term of pH on activation energy was shown to be significant (p-value < 0.05) (Additional file 1: Table S6). Buffer strength and mannitol were the main parameters affecting the activation energy. On the other hand, the buffer strength was found to significantly affect the pre-exponential factor (p-value < 0.05) (Additional file 1: Table S7).

**Fig. 7:**
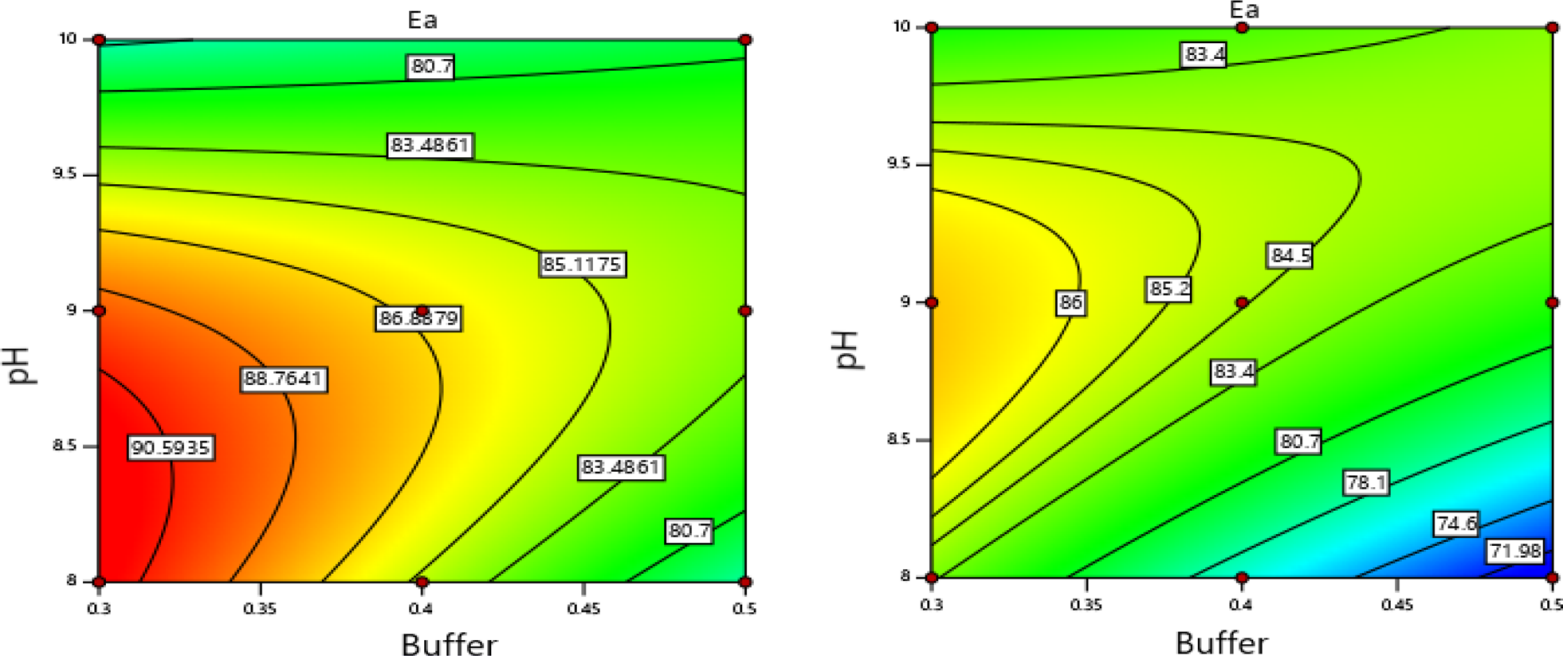
Contour plots for activation energy at a low level of mannitol (left) and at a high level of mannitol (right)

**Fig. 8:**
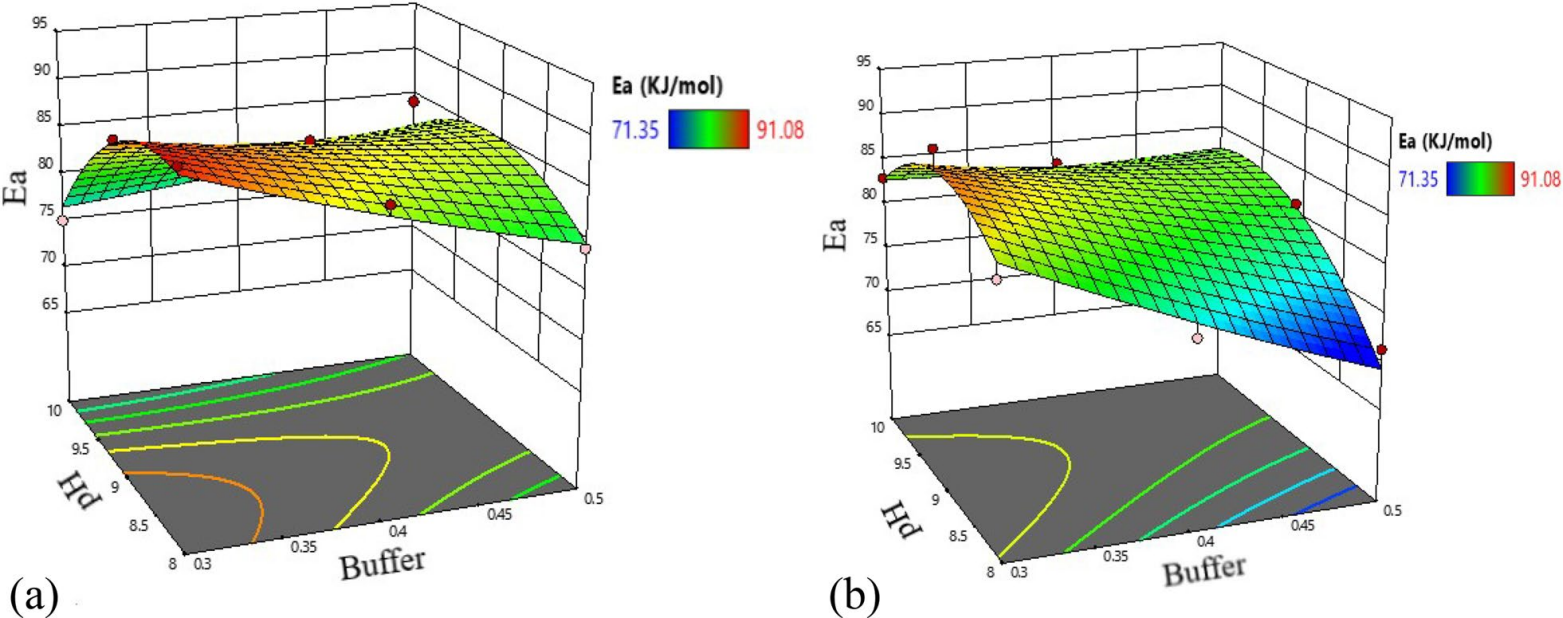
Response surfaces of activation energy based on a low (**a**) and a high (**b**) level of mannitol

Response surface plots were developed for activation energy using the fitted quadratic polynomial equation and were used to locate the points of maximum activation energy. The 2D contour plots and the 3D response surface plots set at a low and high level of mannitol are shown in Figs. 7 and 8. The interaction effects of the factors were observed for activation energy. Figure 7 showed that a change in pH value from 10 to 8 with a decrease in buffer strength from 0.5 to 0.3 M increased the activation energy. Moreover, the contour plots revealed optima in the left section of the plots in the red area covering a pH of 8—9 and a low buffer strength of 0.3 M. Similarly, the 3D response surface plots showed a decrease in activation energy when buffer strength increased above 0.3 M phosphate or deviated from pH 9 mainly to a high level (Fig. 8).

## Discussion

A HPLC method using superficially porous particles (also known as fused-core, porous-shell, core–shell, solid-core) was developed to analyse artesunate efficiently and in a more environmentally friendly manner, emphasizing on the monitoring of its conversion to its main hydrolytic degradation product. The method was found to be suitable to assay artesunate in the presence of its main degradant DHA. Only some methods have been so far developed for the determination of artesunate in bulk and tablet dosage forms [31, 32], and in rectal gel [33], but there was no stability-indicating method yet available for the determination of artesunate in aqueous i.v. solution. Hence, besides its present use in this study, the proposed method can be further applied for stability-indicating analysis of artesunate in liquid formulations because a simple, reliable and economical analytical method is essential to determine the quality of anti-malarial drugs [34].

Hydrolytic degradation is a common pathway of degradation for drugs with ester functional groups [35, 36]. Artesunate was similarly hydrolysed in aqueous solution in the presence of phosphate and mannitol with pseudo-first-order kinetics. The linear relationship between the natural logarithm of the rate constant and the reciprocal of the storage temperature revealed the suitability of the Arrhenius equation (r = 0.9873) to predict the stability of artesunate. By applying the Arrhenius plot, the maximum predicted value of activation energy of the artesunate formulation was 91.08. The reported values of activation energy for most drugs are in the range of 40–130 kJ/mol with an average value of about 80 kJ/mol [37]. This depicts the highest activation energy recorded for artesunate was above the average and with this value, its predicted shelf life (7.30 h at 30 °C) could be longer than the suggested shelf life of the recently available artesunate product after reconstitution [23].

Based on the data from the kinetics study, the rate of hydrolysis of the drug was found to be increased approximately by 9.3 folds as the storage temperature of the formulation was raised by 15–20 ℃. Similarly, it was predicted using the Arrhenius equation, showing a 10 ℃ increase of the temperature accelerated on average 3.4 times the rate of degradation of the drug. This is slightly higher than the rate of hydrolysis of several organic compounds containing ester, carbamate, amide, and lactam functional moieties, which have been reported in the literature [38]. Hence, artesunate hydrolytic degradation could occur at a rapid rate when the storage temperature escalates. As a result of this evidence, it is advisable that the aqueous solution of the product is handled at lower temperatures, preferably in refrigerated conditions (5 ℃) after reconstitution during clinical use.

The analysis of the effects of the buffer strength, pH, and mannitol on the hydrolysis rate constants and Arrhenius parameters using ANOVA showed a significant variation in the responses as the factors changed over the tested levels (p < 0.05). Buffer strength seemed to be the main factor determining the stability of the drug in aqueous solution. Similarly, setting the proper pH of the product could have an important role in stabilizing the drug. These findings are in line with previous studies stating that buffer type, as well as pH, can influence drugs that are liable to hydrolysis [39, 40]. On the other hand, the presence of mannitol in the formulations did not appear to enhance the stability of the drug, as it showed a marked negative impact on the activation energy, and also tended to increase its hydrolysis rate constants. Therefore, mannitol is not suggested to be used as a stabilizing agent in artesunate formulation with phosphate buffer.

Even if a decrease in buffer strength showed slightly a significant increasing effect on the pre-exponential factor, a more pronounced effect was observed on activation energy and hydrolysis rate constants. Hence, the use of diphosphate buffer at a low level likely exhibited a more stabilizing effect toward artesunate in aqueous solution as a considerable increasing value of activation energy and a reduction in the rate constants with a decrease in buffer strength was demonstrated. This could point out the catalytic effect of the buffer and the dependence of the degradation rate of the drug on the phosphate buffer concentration [41]. Moreover, the change in pH of the formulation within the experimental range had an influence on the activation energy, signifying its effect on the stability of the drug, but less compared to the effects of the buffer strength. Generally, the artesunate formulation is revealed to be more stable at a low level of buffer strength and a pH value of not greater than nine.

## Conclusion

The proposed HPLC method was found to be useful for stability indicative assay of artesunate in the development of an i.v. formulation. The stability of artesunate in aqueous solution can be enhanced through formulation using phosphate buffer at optimal values of buffer strength and pH. However, the addition of mannitol into the formulation did not increase the activation energy, indicating that the use of mannitol as a stabilizing agent in artesunate aqueous solution is worthless. Thus, by applying the Arrhenius temperature model and the experimental design, the hydrolysis kinetics can be predicted allowing to perform product development.

## Supplementary Information

**Additional file 1: Table S1**. Experimental design. **Table S2**. The effects of the factors on the hydrolysis rate constants and Arrhenius parameters for each formulation. **Table S3**. Summary of ANOVA results of K at 5 °C. **Table S4**. Summary of ANOVA results of K at 25 °C. **Table S5**. Summary of ANOVA results of K at 40 °C. **Table S6**: Summary of ANOVA results of quadratic models for activation energy. **Table S7**: Summary of ANOVA results of quadratic models for frequency factor.

## Data Availability

The datasets used and/or analysed during the current study are available from the corresponding author upon rational request.
