# Supplementary material for: Degradation kinetics of artesunate for the development of an ex-tempore intravenous injection
Source: Malar J. 2022 Sep 6;21:256. doi: 10.1186/s12936-022-04278-4 (PMC9450271; doi:10.1186/s12936-022-04278-4)
Supplement: Supplementary file 1 — Additional file 1: Table S1. Experimental design. Table S2. The effects of the factors on the hydrolysis rate constants and Arrhenius parameters for each formulation. Table S3. Summary of ANOVA results of K at 5 °C. Table S4. Summary of ANOVA results of K at 25 °C. Table S5. Summary of ANOVA results of K at 40 °C. Table S6: Summary of ANOVA results of quadratic models for activation energy. Table S7: Summary of ANOVA results of quadratic models for frequency factor. [file 12936_2022_4278_MOESM1_ESM.docx]

##### Degradation kinetics of artesunate for the development of an ex-tempore intravenous injection

**Supplementary materials**

**Table S1. Experimental design**

| **Experiment** | **Factors** | | |
| --- | --- | --- | --- |
|  | **Buffer strength** | **Ph** | **Mannitol concentration** |
| 1 | -1 | -1 | -1 |
| 2 | -1 | -1 | +1 |
| 3 | -1 | 0 | -1 |
| 4* | -1 | 0 | +1 |
| 5 | -1 | +1 | -1 |
| 6 | -1 | +1 | +1 |
| 7 | 0 | -1 | -1 |
| 8 | 0 | -1 | +1 |
| 9 | 0 | 0 | -1 |
| 10 | 0 | 0 | +1 |
| 11 | 0 | +1 | -1 |
| 12 | 0 | +1 | +1 |
| 13 | +1 | -1 | -1 |
| 14 | +1 | -1 | +1 |
| 15 | +1 | 0 | -1 |
| 16 | +1 | 0 | +1 |
| 17 | +1 | +1 | -1 |
| 18 | +1 | +1 | +1 |

**Table S2. The effects of the factors on the hydrolysis rate constants and Arrhenius parameters for each formulation**

| **Factors** | | | | **Responses** | | | |
| --- | --- | --- | --- | --- | --- | --- | --- |
| **Buffer (M)** | **pH** | **Mannitol**  **(mmol/mL)** | **K**  **(5 ℃)** | **k**  **(25 ℃)** | **k**  **(40 ℃)** | **Ea**  **(kJ/mol)** | **A**  **(h^-1^)** |
| 0.3 | 8 | 0 | 0.00067 | 0.0054 | 0.0597 | 91.08 | 7.30E+13 |
| 0.3 | 8 | 0.22 | 0.00067 | 0.0056 | 0.0390 | 83.26 | 2.65E+12 |
| 0.3 | 9 | 0 | 0.00075 | 0.0054 | 0.0588 | 88.50 | 2.63E+13 |
| 0.3 | 9 | 0.22 | 0.00066 | 0.0060 | 0.0563 | 90.84 | 6.67E+13 |
| 0.3 | 10 | 0 | 0.00132 | 0.0053 | 0.0553 | 75.01 | 1.29E+11 |
| 0.3 | 10 | 0.22 | 0.00071 | 0.0061 | 0.0400 | 82.89 | 2.45E+12 |
| 0.4 | 8 | 0 | 0.00075 | 0.0051 | 0.0510 | 85.57 | 7.52E+12 |
| 0.4 | 8 | 0.22 | 0.00115 | 0.0065 | 0.0460 | 75.09 | 1.27E+11 |
| 0.4 | 9 | 0 | 0.00077 | 0.0050 | 0.0525 | 86.51 | 1.15E+13 |
| 0.4 | 9 | 0.22 | 0.00081 | 0.0064 | 0.0500 | 84.21 | 4.74E+12 |
| 0.4 | 10 | 0 | 0.00083 | 0.0049 | 0.0504 | 61.49 | 2.90E+8 |
| 0.4 | 10 | 0.22 | 0.00082 | 0.0060 | 0.0472 | 82.67 | 2.44E+12 |
| 0.5 | 8 | 0 | 0.00089 | 0.0061 | 0.0433 | 79.15 | 5.86E+11 |
| 0.5 | 8 | 0.22 | 0.00137 | 0.0070 | 0.0459 | 71.35 | 3.03E+10 |
| 0.5 | 9 | 0 | 0.00090 | 0.0069 | 0.0515 | 82.44 | 2.48E+12 |
| 0.5 | 9 | 0.22 | 0.00082 | 0.0062 | 0.0433 | 81.17 | 1.30E+12 |
| 0.5 | 10 | 0 | 0.00089 | 0.0062 | 0.0555 | 84.14 | 4.87E+12 |
| 0.5 | 10 | 0.22 | 0.00105 | 0.0065 | 0.0567 | 80.99 | 1.46E+12 |

**Table S3. Summary of ANOVA results of K at 5 ℃**

| **Source** | **Sum squares** | **DF** | **Mean square** | **F-value** | **P-value** | **Remarks** |
| --- | --- | --- | --- | --- | --- | --- |
| Model | 5.543E-07 | 6 | 9.238E-07 | 4.50 | 0.0182 | S |
| Buffer(X_1_) | 1.232E-07 | 1 | 1.232E-07 | 6.01 | 0.0342 | S |
| pH(X_2_) | 1.990E-07 | 1 | 1.990E-07 | 9.70 | 0.0110 | S |
| Mannitol (X_3_) | 6.390E-08 | 1 | 6.390E-08 | 3.11 | 0.1081 | NS |
| X_1_X_2_ | 1.275E-07 | 1 | 1.275E-07 | 6.21 | 0.0318 | S |
| X_1_X_3_ | 1.323E-07 | 1 | 1.323E-07 | 6.45 | 0.0294 | S |
| X_2_X_2_ | 1.806E-07 | 1 | 1.806E-07 | 8.80 | 0.0141 | S |
| Residual | 2.052E-07 | 10 | 2.052E-08 |  |  |  |
| Core Total | 7.595E-07 | 16 |  |  |  |  |

S: significant; NS: not significant; P- value of less than 0.5 is considered to be significant

**Table S4. Summary of ANOVA results of K at 25 ℃**

| **Source** | **Sum squares** | **DF** | **Mean square** | **F-value** | **P-value** | **Remarks** |
| --- | --- | --- | --- | --- | --- | --- |
| Model | 3.596E-06 | 3 | 1.199E-06 | 7.25 | 0.0042 | S |
| Buffer(X_1_) | 2.168E-06 | 1 | 2.168E-06 | 13.11 | 0.0031 | S |
| pH(X_2_) | 5.172E-10 | 1 | 5.172E-10 | 0.0031 | 0.9562 | NS |
| Mannitol (X_3_) | 1.415E-06 | 1 | 1.415E-06 | 8.56 | 0.0118 | S |
| Residual | 2.149E-06 | 13 | 1.653E-07 |  |  |  |
| Core Total | 5.745E-06 | 16 |  |  |  |  |

S: significant; NS: not significant

**Table S5. Summary of ANOVA results of K at 40 ℃**

| **Source** | **Sum squares** | **DF** | **Mean square** | **F-value** | **P-value** | **Remarks** |
| --- | --- | --- | --- | --- | --- | --- |
| Model | 0.0004 | 6 | 0.0001 | 2.67 | 0.0821 | NS |
| Buffer(X_1_) | 0.0001 | 1 | 0.0001 | 4.23 | 0.0668 | NS |
| pH(X_2_) | 0.0001 | 1 | 0.0001 | 2.15 | 0.1994 | NS |
| Mannitol (X_3_) | 0.0000 | 1 | 0.0000 | 0.975 | 0.3465 | NS |
| X_1_X_2_ | 0.0001 | 1 | 0.0001 | 3.36 | 0.0969 | NS |
| X_1_X_3_ | 0.0001 | 1 | 0.0001 | 3.73 | 0.0822 | NS |
| X_2_X_3_ | 1.105E-08 | 1 | 1.105E-08 | 0.0004 | 0.9839 | NS |
| Residual | 0.0003 | 10 | 0.0000 |  |  |  |
| Core Total | 0.0007 | 16 |  |  |  |  |

S: significant; NS: not significant

**Table S6: Summary of ANOVA results of quadratic models for activation energy**

| **Source** | **Sum squares** | **DF** | **Mean square** | **F-value** | **P-value** | **Remarks** |
| --- | --- | --- | --- | --- | --- | --- |
| Model | 423.75 | 8 | 52.97 | 11.28 | 0.0013 | S |
| Buffer(X_1_) | 55.27 | 1 | 55.27 | 11.77 | 0.0090 | S |
| pH(X_2_) | 53.51 | 1 | 53.51 | 11.39 | 0.0097 | S |
| Mannitol (X_3_) | 56.03 | 1 | 56.03 | 11.93 | 0.0086 | S |
| X_1_X_2_ | 120.67 | 1 | 120.67 | 25.69 | 0.0010 | S |
| X_1_X_3_ | 17.81 | 1 | 17.81 | 3.79 | 0.0874 | NS |
| X_2_X_3_ | 89.42 | 1 | 89.42 | 19.03 | 0.0024 | S |
| X_1_^2^ | 0.7731 | 1 | 0.7731 | 0.1646 | 0.6956 | NS |
| X_2_^2^ | 88.15 | 1 | 88.15 | 18.77 | 0.0025 | S |
| Residual | 37.58 | 8 | 4.70 |  |  |  |
| Total | 461.32 | 16 |  |  |  |  |

S: significant; NS: not significant

**Table S7: Summary of ANOVA results of quadratic models for frequency factor**

| **Source** | **Sum squares** | **DF** | **Mean square** | **F-value** | **P-value** | **Remarks** |
| --- | --- | --- | --- | --- | --- | --- |
| Model | 2.147E+27 | 1 | 2.147E+27 | 5.34 | 0.0355 | S |
| Buffer(X_1_) | 2.147E+27 | 1 | 2.147E+27 | 5.34 | 0.0355 | S |
| Residual | 6.035E+27 | 15 | 4.024E+26 |  |  |  |
| Total | 8.182E+27 | 16 |  |  |  |  |

S: significant; NS: not significant
